# Summerings

**Published:** 1868-06

**Authors:** Carl Benson


### SUMMERINGS.

*To the Editors of the Evening Post :*

Under this head you insert from *Hall's Journal of Health* some "specimens of laconic good sense." It has not occurred to you that these rules, however excellent in themselves, are a positive plagiary from the *Bunkum Flagstaff*, which recently published a set of similar rules. As all your readers may not have seen the *Flagstaff's* duodecalogue, I take the liberty of enclosing a copy. CARL BENSON.

"SUMMARY SUMMERINGS.

"1. When you go out of town resolve to sink the shop. This may be a difficult thing to do if you have been talking shop all your life ; therefore, suppose you try the experiment for a little while of not talking at all. It will be a pleasing variety, and may give you the reputation of a great thinker.

"2. City officials have brought the term gentleman into bad odor. Therefore don't call yourself anything of the sort. Say at once that you are a loafer ; stick your feet into the windows and spit. It saves no end of trouble, and also enables you to fraternize easily with a large and valuable portion of the indigenous population.

"3. Don't say you know an alderman, lest people lock up their plated spoons ; or an editor, lest they think you are a correspondent and going to put them into his paper (country folks are so conceited); or an artist, lest some donkey set you down for a dissipated Bohemian.

"4. As a general rule it is safest not to know anybody or anything ; then you cannot be suspected of knowing more than your neighbors, which is very anti-republican.

"5. On starting for the country you will be puzzled what to do with your "Sunday-go-to-meeting" clothes. If you leave them behind, the moths, which as an observant foreigner has lately remarked, really seem to like camphor, cedar and tobacco, will most probably make away with them ; if you take them with you, you will waste their sweetness on the desert natives, who will morever be annoyed by your appearing in better rig than their own. In this dilemma, your best plan is to have no good clothes. Buy you habiliments at a slop-shop, and wear them as long as they will stick together. Thus you will have money (a great point now-a-days,) and avoid the dangerous reputation of being "stuck up."

"6. However bad any food may be, don't complain of it. Remember that an American citizen has no right to find fault with anything, and that if your landlord chisels you he is only following the example of most great railroad and steamboat companies. Remember, also, that if the son of your loins and the daughter of your bosom ever know enough to keep a hotel, or take boarders, they will be able to impose upon the public in like manner. Therefore, if the viands offered you are utterly uneatable, all you have to do is not to eat them. By fasting for three days you will probably become hungry enough to eat an old boot without sauce, or a cat without stopping to cut the claws off.

"7. You don't go into the country to eat; else you would be wonderfully disappointed. Nor do you go to dress; therefore dress as little as possible. If it is hot, walk about in your shirt and continuations like the plain farmers of the vicinity. Go barefoot if the roads are not too gravelly. It saves expense, and makes you a favorite with the ladies.

"8. When you are at Rome do as the Romans do, however vain they may be. Conform to the manners and customs of the people about you, provided such conformity does not lead straight to the hospital or Penitentiary. Thus, if the aborigines drink whiskey warranted to kill at fifty yards, you may decline joining them on the plea of being subject to pericarditis or a total temperance society; but if they habitually speak bad grammar, by all means do the same. It is an innocent concession to their prejudices, and bad English is nowhere forbidden in Scripture.

"9. Be cheerful, be kind, be considerate. Of course you have no occasion for these virtues in town. They might interfere with your business and derogate from your independence. But in the country, where nobody knows you, you may indulge yourselves and others in these unusual practices.

"10. It is best not to have any politics or religion; then you cannot offend persons of the opposite ones.

"11. Never argue, unless you are going through a course of Banting, and even then a walk of ten miles is better. Argument never does anybody else any good, and it will do you none unless you are paid for it. Leave it to the editors and politicians and lawyers, who are paid for it.

"If you are unfortunate enough to have a particular religion, the next best thing is to use it as little as possible. A safe rule is, "Always go against your own church and never into any."—[*Bunkum Flagstaff*, *issue April* 1*st*.

